# Functional analysis and regulation of purified connexin hemichannels

**DOI:** 10.3389/fphys.2014.00071

**Published:** 2014-02-25

**Authors:** Mariana C. Fiori, Luis Reuss, Luis G. Cuello, Guillermo A. Altenberg

**Affiliations:** Department of Cell Physiology and Molecular Biophysics, Center for Membrane Protein Research, Texas Tech University Health Sciences CenterLubbock, TX, USA

**Keywords:** purification, method, gap junction, phosphorylation, PKC, calcium, ATP, permeability

## Abstract

Gap-junction channels (GJCs) are aqueous channels that communicate adjacent cells. They are formed by head-to-head association of two hemichannels (HCs), one from each of the adjacent cells. Functional HCs are connexin hexamers composed of one or more connexin isoforms. Deafness is the most frequent sensineural disorder, and mutations of Cx26 are the most common cause of genetic deafness. Cx43 is the most ubiquitous connexin, expressed in many organs, tissues, and cell types, including heart, brain, and kidney. Alterations in its expression and function play important roles in the pathophysiology of very frequent medical problems such as those related to cardiac and brain ischemia. There is extensive information on the relationship between phosphorylation and Cx43 targeting, location, and function from experiments in cells and organs in normal and pathological conditions. However, the molecular mechanisms of Cx43 regulation by phosphorylation are hard to tackle in complex systems. Here, we present the use of purified HCs as a model for functional and structural studies. Cx26 and Cx43 are the only isoforms that have been purified, reconstituted, and subjected to functional and structural analysis. Purified Cx26 and Cx43 HCs have properties compatible with those demonstrated in cells, and present methodologies for the functional analysis of purified HCs reconstituted in liposomes. We show that phosphorylation of serine 368 by PKC produces a partial closure of the Cx43 HCs, changing solute selectivity. We also present evidence that the effect of phosphorylation is highly cooperative, requiring modification of several connexin subunits, and that phosphorylation of serine 368 elicits conformational changes in the purified HCs. The use of purified HCs is starting to provide critical data to understand the regulation of HCs at the molecular level.

## Introduction

**Figure 1:**
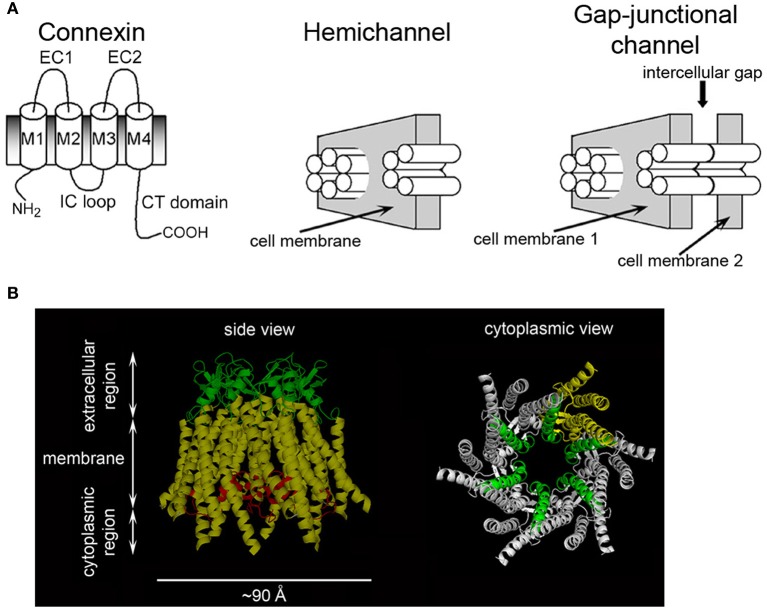
**Connexins, hemichannels, and gap-junctional channels**. **(A)** Representation of a connexin, a HC and a GJC. M1-M4: trans-membrane helices; EC1 and EC2: extracellular loops; IC loop: intracellular loop. Modified from Bao et al. (2005). **(B)** Cx26 HC structure. Left: transmembrane helices (yellow), EC loops (green), and N-terminal region (red). Part of the latter forms a short α helix that inserts into the pore and has a role in voltage gating. Right: HC view showing M1 (green) lining the pore throughout. M2, M3, and M4 from one subunit are shown in yellow. The N-terminal region was removed to facilitate observation of M1.

Gap-junction channels (GJCs) formed by connexins are responsible for cell-to-cell communication in eukaryotic cells (Mese et al., 2007; Nielsen et al., 2012; Abascal and Zardoya, 2013). GJCs are formed by head-to-head docking of two connexin hexamers referred to as hemichannels (HCs) or connexons, one from each of the neighboring cells (Figure 1A). GJCs and HCs are permeable to large hydrophilic solutes, depending on their isoform composition (Harris, 2001; Nielsen et al., 2012). There are 21 human connexin isoforms, varying in length from 226 to 543 amino acids, which display diverse properties in terms of solute permeabilities, regulation and associations with other proteins (Hua et al., 2003; Mese et al., 2007; Nielsen et al., 2012; Abascal and Zardoya, 2013). Figure 1A shows a representation of a connexin, a HC and a GJC. Connexins have four transmembrane α helices (M1-M4) that extend a significant distance outside the plasma membrane. The N- and C-terminal ends and the intracellular loop are intracellular. The available crystal structure of a Cx26 HC in Figure 1B shows M1 and M2 as the primary and secondary pore-lining helices, respectively, with the narrowest region of the pore near the extracellular surface of the membrane (Maeda et al., 2009; Nielsen et al., 2012). The extracellular loops, which contain six conserved cysteines that form intramolecular disulfide bonds, have an essential role in HC docking (Foote et al., 1998; Bao et al., 2004b). The primary sequence of the intracellular loop is not well conserved (Hua et al., 2003; Abascal and Zardoya, 2013); this region seems to interact with the C-terminal domain (CTD) during the regulation of HCs by intracellular acidification (Delmar et al., 2004; Hirst-Jensen et al., 2007). The N-terminal region has a role in voltage gating (Purnick et al., 2000; Gonzalez et al., 2007; Bargiello et al., 2012; Kronengold et al., 2012), whereas the CTD displays large variations in length among connexin isoforms and is involved in regulation and protein-protein interactions (Francis et al., 1999; Hua et al., 2003; Agullo-Pascual and Delmar, 2012; Herve et al., 2012; Abascal and Zardoya, 2013). Cx26 and Cx43 are two of the most different connexins regarding their primary sequence and length of the C-terminal sequence, which comprises ~20 amino acids in Cx26 and ~150 in Cx43.

GJCs are aqueous channels that communicate two compartments that normally have a very similar composition (the cytoplasms of adjacent cells), and are formed by head-to-head association of two HCs, one from each of the adjacent cells. HCs that are “free” at the plasma membrane (not forming GJCs) communicate two compartments of very different composition (intracellular and extracellular fluids). Whereas GJCs are mostly open and mediate electrical and chemical coupling between cells, HCs at the plasma membrane have a low open probability (Po) due, at least in part, to the combination of the high, cell-negative, membrane voltage, and the millimolar concentration of extracellular Ca^2+^ (Li et al., 1996; Contreras et al., 2003; Gonzalez et al., 2007; Fasciani et al., 2013). However, they still play roles in physiologic processes by mediating the transmembrane fluxes of hydrophilic molecules such as ATP, NAD^+^, glutamate, glutathione, PGE_2_ and glucose (Bruzzone et al., 2001; Ye et al., 2003; Cherian et al., 2005; Rana and Dringen, 2007; Retamal et al., 2007a; Kang et al., 2008; Orellana et al., 2011; Wang et al., 2013). Sustained opening of HCs under pathological conditions may result in water and solute fluxes that cannot be compensated by the cells. These fluxes include metabolite loss, Ca^2+^ influx, equilibration of ionic gradients and cell swelling and lead to cell damage. Normally, the combination of the cell-membrane voltage and millimolar concentrations of extracellular divalent cations keep HC Po low (Contreras et al., 2003; Bukauskas and Verselis, 2004; Chen et al., 2005; Sanchez et al., 2010; Fasciani et al., 2013). However, Cx43 HCs can be activated in the presence of extracellular divalent cations under a number of conditions including ischemia/hypoxia (John et al., 1999; Li and Nagy, 2000; Li et al., 2001; Contreras et al., 2002; Vergara et al., 2003b; Saez et al., 2005; Shintani-Ishida et al., 2007). Studies in cardiomyocytes, astrocytes, and renal proximal tubule cells strongly suggest that Cx43 HCs are activated by ATP depletion and that the mechanism may involve dephosphorylation and oxidation (John et al., 1999; Li and Nagy, 2000; Li et al., 2001; Contreras et al., 2002; Vergara et al., 2003b; Saez et al., 2005; Retamal et al., 2006; Shintani-Ishida et al., 2007).

## Connexin 26

Cx26 is expressed in a number of tissues, and its mutations are frequently associated with deafness and skin diseases (Forge and Wright, 2002; Forge et al., 2003; Gerido and White, 2004; Zhao et al., 2006; Nickel and Forge, 2008; Lee and White, 2009; Liu et al., 2009). Here we will focus on the role of Cx26 in the inner ear. In the inner ear, the cochlea houses the organ of Corti, a narrow spiral of epithelial sensory cells (hair cells) that transduces sound waves into electrical impulses. The cochlear gap-junctional communication network includes several different cell types and is essential for hearing (Steel, 1999; Forge and Wright, 2002; Forge et al., 2003; Wangemann, 2006; Nickel and Forge, 2008). Profound hearing loss of genetic origin is common (~1 in 2000 children) and mutations of Cx26, the main connexin in the inner ear, are its major cause (Steel, 2000; Ravecca et al., 2005; Sabag et al., 2005; Apps et al., 2007; Nickel and Forge, 2008; Lee and White, 2009; Martinez et al., 2009; Laird, 2010; Terrinoni et al., 2010; Zoidl and Dermietzel, 2010).

The receptor function of the hair cells requires high [K^+^] in the endolymph and a positive endolymphatic electrical potential (Steel, 1999; Forge and Wright, 2002; Forge et al., 2003; Teubner et al., 2003; Wangemann, 2006; Zhao et al., 2006; Nickel and Forge, 2008). Both are generated by K^+^ transport by the *stria vascularis* and depend on an intact gap-junctional system (Steel, 1999; Forge and Wright, 2002; Forge et al., 2003; Wangemann, 2006; Nickel and Forge, 2008). Activation of the hair cells by sound waves opens non-selective cation channels near the tips of the stereocilia, eliciting influxes of Ca^2+^ and K^+^ and membrane depolarization (Kikuchi et al., 2000; Steel and Kros, 2001; Forge and Wright, 2002; Forge et al., 2003; Nickel and Forge, 2008). It has been proposed that the K^+^ that enters the hair cells moves across their basolateral membrane into the perilymphatic space between hair cells and supporting cells. This K^+^ is then taken up by the supporting cells and recycled back into the endolymph *via* the cochlear gap-junctional network, moving from the supporting cells through the root cells, spiral ligament fibroblasts and the *stria vascularis*, from where it is secreted back to the *scala media* endolymph (Spicer and Schulte, 1998; Steel, 1999; Kikuchi et al., 2000; Forge and Wright, 2002; Forge et al., 2003; Zhao et al., 2006; Nickel and Forge, 2008; Liu et al., 2009). Absence of Cx26 results in death of the hair cells, but the mechanism is unknown. It has been speculated that deafness is a consequence of decreased K^+^ recycling in the cochlea (Johnstone et al., 1989; Kudo et al., 2003; Wangemann, 2006). However, the notion of cochlear K^+^ recycling has been questioned (Patuzzi, 2011), and there are deafness-associated Cx26 mutants that form K^+^ permeable GJCs, but show more subtle permeability changes, such as decrease in pore size or changes in charge-selective permeability (Goldberg et al., 1999, 2002; Bruzzone et al., 2003; Beltramello et al., 2005; Chen et al., 2005; Zhao, 2005; Deng et al., 2006; Anselmi et al., 2008; Gossman and Zhao, 2008; Mese et al., 2008; Majumder et al., 2010). In this case (e.g., Cx26 V84L), the mutations may affect second messenger transport between cells.

It has also been proposed that “leaky” mutant HCs can lead to cell damage and deafness (Stong et al., 2006; Gerido et al., 2007; Lee et al., 2009). Water and solute fluxes through mutant “leaky” HCs (metabolite loss, Ca^2+^ influx, equilibration of ionic gradients, cell swelling) can lead to cell damage and deafness. It has also been speculated that ATP in the endolymph is essential to maintain healthy hair cells, and deafness could result from Cx26 mutations that decrease HC-mediated ATP secretion (Anselmi et al., 2008; Majumder et al., 2010). Recent observations suggest that ATP released through Cx26 and Cx30 HCs on the endolymphatic side of supporting cells activates purinergic receptors in inner hair cells, evoking bursts of action potentials in the developing organ of Corti (Gale et al., 2004; Anselmi et al., 2008; Majumder et al., 2010). Ca^2+^ signaling seems to be essential to maintain healthy hair cells, and therefore alterations in signaling may have an important role in genetic deafness. A role of cochlear Ca^2+^ signaling in the response to damaging stimuli has also been proposed, and an increase in Ca^2+^ permeability through HCs formed by a Cx26 mutant associated with keratitis ichthyosis deafness syndrome (Cx26 G45E) has been identified (Sanchez et al., 2010).

## Connexin 43

Cx43 is the most ubiquitous connexin isoform. Its CTD contains a number of domains involved in interactions with a variety of proteins such as tubulin, tyrosine kinases, ubiquitin ligase, zonula occludens 1 (ZO-1) and Na^+^ channels (Warn-Cramer and Lau, 2004; Solan and Lampe, 2009; Agullo-Pascual and Delmar, 2012; Herve et al., 2012; Agullo-Pascual et al., 2013). Here, we will focus on the role of Cx43 in the cardiovascular system.

Mutations of Cx43 are associated with arrhythmias, oculodentodigital dysplasia, and other genetic disorders, and alterations in Cx43 expression and function play important roles in the pathophysiology of frequent medical problems such as those related to cardiac and brain ischemia as well as wound healing in diabetes (Solan and Lampe, 2009; Eugenin et al., 2012; Marquez-Rosado et al., 2012; Orellana et al., 2012; Churko and Laird, 2013; Giaume et al., 2013). Cx43 is by far the most abundant heart connexin, expressed at high levels in atrial and ventricular myocytes, and to a lower extent in parts of the ventricular conduction system (Fontes et al., 2012). Normally, Cx43 is located at the intercalated discs and its density is low at the lateral membranes (Miura et al., 2010; Duffy, 2012; Fontes et al., 2012; Jeyaraman et al., 2012; Remo et al., 2012). In combination with other factors, this distribution results in a faster longitudinal conduction velocity that is the basis for anisotropic conduction. The preferential location of Cx43 at the intercalated discs depends on a number of factors, including its association with ZO-1 (Remo et al., 2012). Cardiac remodeling occurs in response to a variety of cardiac disorders, and is characterized by structural and electrical alterations that decrease heart electrical stability (Miura et al., 2010; Duffy, 2012; Fontes et al., 2012). The changes in impulse conduction are associated with the phenomenon of lateralization, defined by decreased Cx43 expression, with a relative increase in lateral *vs*. intercalated-disc Cx43 expression, which is often associated with abnormal conduction and arrhythmias (Miura et al., 2010; Duffy, 2012; Fontes et al., 2012; Jeyaraman et al., 2012; Remo et al., 2012). Lateralization is observed in a variety of acquired and inherited arrhythmic syndromes, including ischemic heart disease, hypertrophic cardiomyopathy and arrhythmogenic right ventricular cardiomyopathy (Miura et al., 2010; Duffy, 2012; Fontes et al., 2012; Remo et al., 2012). Although the events leading to lateralization are not fully understood, it seems that under ischemia or conditions of cell stress, the tyrosine kinase Src is activated and binds to ZO-1, competing with the Cx43 binding site, which releases Cx43 from ZO-1. As a result, ZO-1 remains at the intercalated discs, while the plaques move to the lateral membrane (Duffy, 2012). Details on the regulation of Cx43 GJCs and HCs are presented later.

## Why study purified hemichannels?

Extensive information has been obtained on GJC and HC expression and function from cell-biology and functional studies in cells and organs in normal and pathological conditions. For example, there is evidence for changes in Cx43 phosphorylation state under ischemia in the heart (Solan et al., 2007; Solan and Lampe, 2009; Miura et al., 2010; Jeyaraman et al., 2012; Marquez-Rosado et al., 2012), and opening of HCs seems to have a role in cardiomyocyte damage in ischemia (John et al., 1999; Li and Nagy, 2000; Li et al., 2001; Contreras et al., 2002; Vergara et al., 2003b; Shintani-Ishida et al., 2007; Hawat et al., 2010). It is also known that HCs open in response to PKC inhibitors (Bao et al., 2004a). However, molecular mechanisms are hard to tackle in these systems because of their inherent complexities. Experiments *in vivo* are fundamental to understand biological processes, but *in vitro* studies using isolated systems under well-controlled conditions are also an essential component for a complete understanding of normal function and the molecular mechanisms of diseases. In this context, studies of purified HCs provide direct structural and conformational information that serves as an essential complement to the studies in more complex system.

HCs have physiological and pathophysiological significance (John et al., 1999; Bruzzone et al., 2001, 2003; Contreras et al., 2002; Vergara et al., 2003a; Ye et al., 2003; Beltramello et al., 2005; Cherian et al., 2005; Stong et al., 2006; Gerido et al., 2007; Rana and Dringen, 2007; Retamal et al., 2007a; Shintani-Ishida et al., 2007; Anselmi et al., 2008; Gossman and Zhao, 2008; Kang et al., 2008; Lee et al., 2009; Hawat et al., 2010), but the information obtained with HCs also contributes to our understanding of GJCs. Cx26 GJCs behave like two HCs in series (Chen et al., 2005; Sanchez et al., 2010), whereas Cx43 HCs expressed in HeLa cells display single-channel events with conductance and kinetics consistent with Cx43 GJCs, even though Cx43 HCs are activated only at very positive intracellular voltages, a feature different from the voltage sensitivity of Cx43 GJCs (Saez et al., 2005). In addition, the mechanisms of GJC and HC regulation overlap very well (Moreno et al., 1992, 1994; Takens-Kwak and Jongsma, 1992; Kwak et al., 1995; Lampe et al., 2000; Bao et al., 2004a,c, 2007; Delmar et al., 2004; Saez et al., 2005; Ek-Vitorin et al., 2006), and therefore studies on the mechanisms of regulation of HCs also contribute to our understanding of GJC regulation.

## Expression/purification and characterization of purified connexins

Recombinant connexins have been expressed in a variety of systems for cell-biology, biochemical and functional studies, notably mammalian cell lines and frog oocytes. For detailed biochemical studies that require purified connexins, the proteins have been expressed in mammalian and insect cells. Although expression in mammalian cells has been used successfully (Koreen et al., 2004), the insect cell/baculovirus expression system is the only one available that yields milligram amounts of purified connexins. This system has been useful to express Cx26 and Cx43, as well as a variety of mutants (Stauffer, 1995; Bao et al., 2004c, 2007; Oshima et al., 2007, 2008; Gassmann et al., 2009; Maeda et al., 2009; Ambrosi et al., 2010; Fiori et al., 2012).

In most cases, we express connexins modified by insertion of a protease cleavage site and a poly-His tag at the C-terminal end (Bao et al., 2004c; Fiori et al., 2012). The protease cleavage sites are selective for either thrombin or TEV proteases. These connexin DNA sequences are cloned into baculovirus transfer vectors and used to generate recombinant baculoviruses. We have used the Invitrogen Bac-to-Bac system successfully. The viruses produced in Sf9 insect cells are used to infect insect cells for protein production, in either Sf9 or High-Five cells working well for connexin expression (Bao et al., 2004c, 2007; Fiori et al., 2012). We generally use Sf9 cells in suspension, grown in serum-free HyClone CCM3 medium supplemented with gentamycin. The cells grown at 26°C are infected (generally 2 viral particles/cell) and the cells are harvested ~2 days after infection, when viability is ~40%. For purification, the cell pellets are resuspended in a 1-mM bicarbonate solution containing 1 mM protease inhibitors, and lysed. The membranes are alkali-extracted by addition of NaOH, then solubilized with 2.5% *n*-dodecyl-β-D-maltoside in the presence of a high salt concentration (2 M NaCl), a chelator of divalent cations, a reducing agent and 10% glycerol. The solubilized material is then purified based on the affinity of the His tag for Co^2+^, followed by size-exclusion chromatography. If needed, removal of the His tag is accomplished by site-specific proteolysis, which is followed by isolation of the untagged connexin by size-exclusion chromatography. The insect-cell/baculovirus expression system yields approximately 0.5 mg/l culture of highly-pure connexins suitable for biochemical, structural and functional studies (Bao et al., 2004c; Fiori et al., 2012).

Connexins expressed in insect cells have been successfully employed for structural studies using X-ray crystallography and cryo-electron microscopy (Hoh et al., 1993; Oshima et al., 2003, 2007, 2008, 2011; Gassmann et al., 2009; Maeda et al., 2009; Ambrosi et al., 2010). Electron-microscopy data point to Cx26 purified as HCs (hexamers), as opposed to GJCs (dodecamers), and our studies agree with that notion (Bao et al., 2004c; Fiori et al., 2012). We have recently performed a detailed biochemical and biophysical characterization of purified Cx26 (Fiori et al., 2012). A single absorbance peak was observed in size-exclusion chromatograms, coincident with the high degree of purity estimated from Coomassie blue-stained gels. The apparent molecular weight of the protein-detergent complex was estimated at 235 kDa, whereas its average hydrodynamic radius determined by dynamic light scattering was 5.4 nm. These values are consistent with a Cx26 hexamer-detergent complex. The purified Cx26 hexamers were highly-structured, with a calculated α-helix content of 59%, in reasonable agreement with the recent crystal structure of Cx26 that shows an approximate α helical content of 54%.

It is interesting to note that the properties of purified Cx26 and Cx43 differ. While Cx26 is purified as HCs that are highly stable (Fiori et al., 2012), purified Cx43 HCs are not stable in detergent, but are stable in lipids (Bao et al., 2007). In detergent and at low concentrations Cx43 is mostly present as monomers, whereas at higher concentrations it forms hexamers that allow for subunit exchange (Bao et al., 2007). The lower stability of Cx43 HCs in solution is very useful because it allows the generation of Cx43 HCs with controlled subunit composition (see below).

## Functional analysis of purified and reconstituted connexin hemichannels

Recombinant purified GJCs and HCs expressed in Sf9 insect cells have been used extensively for structural studies. In contrast, functional studies are few, and have been performed without control of a number of variables known to affect HC gating, such as transmembrane voltage and redox state (Retamal et al., 2006, 2007b; Gonzalez et al., 2007; Bargiello et al., 2012). Functional assays for purified connexin HCs can be generally divided into qualitative assays used to determine whether HCs are permeable to a solute or not, and quantitative/semi-quantitative assays that are more suitable to determine changes in HC permeability properties. The former include the transport-specific fractionation technique developed by Harris and collaborators (Harris et al., 1989; Harris and Bevans, 2001; Bao et al., 2004c, 2007; Fiori et al., 2012), and probe-permeation assays that use labeled solutes to determine whether they can enter into liposomes containing HCs or be released from liposomes pre-loaded with the probe (Bevans et al., 1998; Bao et al., 2004a; Fiori et al., 2012). The latter includes single-channel electrophysiological studies and assays of solute influx into liposomes containing HCs (Buehler et al., 1995; Rhee et al., 1996; Gassmann et al., 2009; Fiori et al., 2012).

**Figure 2:**
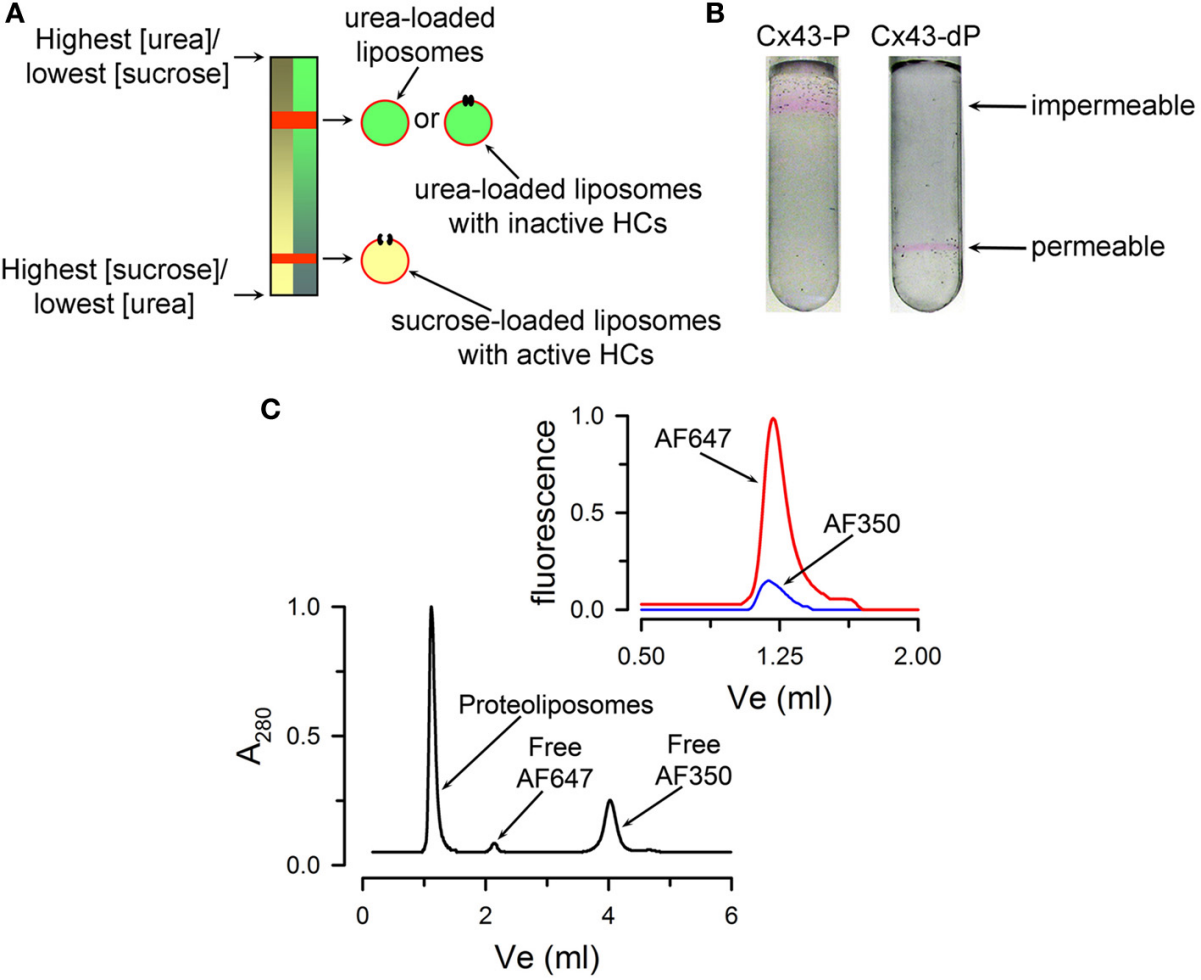
**Qualitative transport assays. (A)** Schematic representation of the transport-specific assay. **(B)** Typical migration of liposomes containing purified Cx43 HCs on an isoosmolar linear sucrose density gradient. Images showing the position of the rhodamine B-labeled proteoliposomes after centrifugation. The HCs were formed by either fully dephosphorylated Cx43 (Cx43-dP, sucrose permeable) or Cx43 fully phosphorylated by PKC (all six Ser-368 residues phosphorylated, Cx43-P, impermeable to sucrose). Reconstitution was at an average HC/liposome ratio of 2.3. **(C)** Permeability of reconstituted HCs to Alexa Fluor probes. Liposomes pre-loaded with Alexa Fluor 350 (AF350) or 647 (AF647) were run on the size-exclusion column to separate free extraliposomal dyes from the dyes inside the liposomes. A_280_ is the absorbance at 280 nm. Top right: AF350 and AF647 fluorescence associated with the liposomes containing Cx26. The data were normalized to the peak emission of parallel experiments with liposomes without Cx26. Panels **(B)** and **(C)** were modified from Bao et al., 2004c and Fiori et al., 2012, respectively.

The transport-specific fractionation technique is used to determine whether HCs are permeable or not to sucrose and other hydrophilic solutes (Harris et al., 1989; Harris and Bevans, 2001; Bao et al., 2004c, 2007; Fiori et al., 2012). Although qualitative, it is a very powerful technique because it depends directly on solute transport and rules out solute binding to the liposomes. The method is based on the migration of liposomes upon centrifugation in a linear isoosmolar sucrose gradient where the concentration of sucrose increases from top to bottom, and the concentration of urea decreases from top to bottom, in such a way that the osmolality remains constant. The liposomes or proteoliposomes containing HCs impermeable to sucrose remain in the upper part of the tube, buoyed up by the entrapped urea solution of lower density. The heavier sucrose-loaded liposomes containing sucrose-permeable HCs migrate as a narrow band to a lower position in the tube. A schematic representation of the technique is shown in Figure 2A. The experiments are simplified by the incorporation into the liposomes of traces of a fluorescent lipid, which allows for easy following of the liposome position in the gradient. An example is shown in Figure 2B. Also, transport-specific fractionation can be used in combination with permeability assays for other solutes. For these studies, the liposomes are pre-loaded with the probes (e.g., radiolabeled or fluorescent permeability probes), and permeability is determined from the retention of the probes in the liposomes (impermeable) or their loss (permeable). The transport-specific fractionation of liposomes is also very useful to determine the fraction of purified connexins that form functional HCs (Rhee et al., 1996; Bao et al., 2004c, 2007), a very important parameter to assess the quality of the preparation. For these studies, HCs reconstitution is done in such a way that the average yield is less than one HC/liposome. Under these conditions, the experimental data (percentage of sucrose-permeable liposomes with one or more functional HCs) can be compared with the prediction based on the protein/lipid ratio and the size of the liposomes. Sucrose-impermeable liposomes will be those without HCs and those with non-functional HCs. Our studies showed that essentially all purified Cx26 and Cx43 HCs purified from insect cells form functional HCs (Bao et al., 2007; Fiori et al., 2012).

A more standard assay for solute permeability is based on probe uptake or efflux. For example, liposomes containing purified Cx26 HCs pre-loaded with Alexa Fluor 350 (349 Da) and Alexa Fluor 647 (1300 Da) can be separated from free probes by size-exclusion chromatography (Fiori et al., 2012). Figure 2C shows a typical size-exclusion chromatogram where liposomes are separated from the extraliposomal free dyes. Alexa Fluor 350 and 647 were retained by the liposomes without HCs, whereas only the latter was retained inside the proteoliposomes with Cx26 HCs. Similar experiments can be performed with physiologically-relevant solutes such as ATP, inositol phosphates and cyclic nucleotides (Ayad et al., 2006; Harris, 2007).

**Figure 3:**
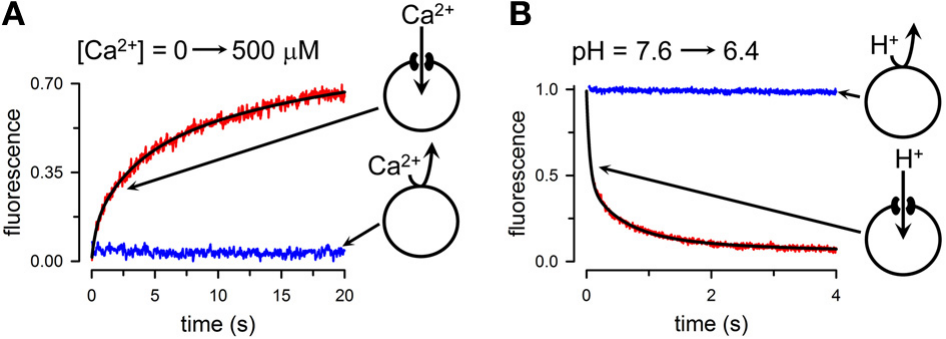
**Quantitative transport assays. (A)** Rate of Ca^2+^ influx into liposomes containing purified Cx26 HCs. Extraliposomal free-[Ca^2+^] was rapidly increased by mixing in a stop-flow cell from a few nM to 500 μM, and the rate of increase in emission from Fluo-5N trapped into the liposomes was followed. Red record: liposomes containing Ca^2+^-permeable Cx26 HCs; blue record: liposomes without HCs. The black line is a multi-exponential fit to the data used to calculate the rate of Ca^2+^ influx. **(B)** Changes in intraliposomal pH followed by the pH-sensitive probe fluorescein attached to a phospholipid head group. Extraliposomal pH was rapidly reduced from 7.6 to 6.4 by mixing in a stop-flow cell, and the rate of decrease in pH was followed by the quenching of the fluorescein bound to the inner leaflet of the liposomes bilayer. The black line is a multi-exponential fit to the data used to calculate the rate of liposome acidification. Modified from Fiori et al., 2012.

Single-channel analysis is the assay of choice to evaluate the permeability properties of connexin HCs to small inorganic ions. Unfortunately, the experience with purified HCs has been mixed at best, with HCs often not showing the single-channel conductance and kinetics expected from the studies in cells (Buehler et al., 1995; Rhee et al., 1996; Gassmann et al., 2009). This is clearly an area that needs additional experimental work. Therefore, for the more quantitative studies, we will focus on assays that we developed recently to study permeation of Ca^2+^, Na^+^, and H^+^. In principle, intercellular Ca^2+^ signaling can be the result of movements through GJCs of second messengers such as IP3 and/or Ca^2+^, but signaling is also possible through a paracrine pathway, by the release of ATP through HCs (Piazza et al., 2007; Anselmi et al., 2008; Kang et al., 2008; Mammano, 2013). In this case, activation of purinergic receptors in neighboring cells elicits Ca^2+^ influx in those cells. Although extracellular [Ca^2+^] at millimolar concentrations decreases HC activity (Li et al., 1996; Contreras et al., 2003; Chen et al., 2005; Fasciani et al., 2013; Lopez et al., 2013), HCs can still be activated at high extracellular [Ca^2+^] under a number of conditions, including ischemia, inflammation, connexin dephosphorylation, and extracellular alkalinization (John et al., 1999; Li and Nagy, 2000; Contreras et al., 2002; Bao et al., 2004a; Retamal et al., 2007b; Shintani-Ishida et al., 2007; Hawat et al., 2010; Schalper et al., 2010; Orellana et al., 2011; Eugenin et al., 2012). In spite of indirect evidence from studies in cells, the possibility of Ca^2+^ movement through HCs and GJCs had not been addressed directly until recently. Reasons include the minimal number of functional studies of purified HCs, and the absence of an assay to follow Ca^2+^ permeation in purified HCs. In order to address whether HCs are permeable to Ca^2+^, we developed an assay to follow the time course of changes in intraliposome [Ca^2+^] that result from influx through HCs, and tested the assay on purified Cx26 HCs (Fiori et al., 2012). Basically, we loaded liposomes with the low-affinity Ca^2+^-sensitive fluorescent probe Fluo-5N, and then removed the extraliposomal probe by gel filtration. Fluo-5N is retained inside the liposomes in the presence or absence of Cx26 HCs because its size and charge (958 Da, -5 net charge). Typical experiments are shown in Figure 3A, where Fluo-5N emission increased following the elevation of free-[Ca^2+^] from <10 nM to 500 μM in the liposomes containing HCs (red trace), but not in liposomes without HCs (blue trace). In these experiments, the increase in extraliposomal [Ca^2+^] was achieved in <0.5 ms in a stop-flow cell. Analysis of the changes in fluorescence allows for estimations of Ca^2+^ influx and permeability. With a similar approach, but using the Na^+^-sensitive fluorescent probe SBFI, we determined that the permeabilities of Cx26 HCs to Na^+^ and Ca^2+^ are similar, pointing to a high Ca^2+^ permeability. In spite of the high Ca^2+^ permeability, since cytosolic [Ca^2+^] and [Ca^2+^] gradients are low, significant cell-to-cell Ca^2+^ fluxes through GJCs will depend critically on the number of permeable channels. In contrast, the much larger electrochemical driving force for Ca^2+^ influx into cells suggests a significant role of HCs in Ca^2+^ influx, at least under certain circumstances (e.g., ischemia), or in disease-causing mutants that display higher Ca^2+^ permeability (G45E mutant associated with keratitis ichthyosis deafness syndrome) (Sanchez et al., 2010).

We have also developed a variation of the methodology above to assess the permeation of H^+^ equivalents (H^+^/OH^−^/buffer transport) (Fiori et al., 2012). For these studies, the liposomes contain traces of a phospholipid labeled with fluorescein at the head group. When extraliposomal pH is reduced from 7.6 to 6.4 in a stop-flow cell, fluorescein emission decreased in the Cx26-HC liposomes exposed to the pH gradient (Figure 3B, red trace), but not in the liposomes without HCs (Figure 3B, blue trace). Upon lowering extraliposomal pH, there is quenching of the fluorescein emission from the outer leaflet of the liposome bilayer. This quenching is independent of the presence of HCs, and is too fast for detection in the stop-flow setup. Only the slower fluorescence quenching that results from the effects of intraliposomal acidification on the inner-leaflet fluorescence is then recorded (Figure 3B). From the pH changes, buffer composition and other parameters, we estimated an H^+^-equivalent permeability ~10-fold higher than that for Na^+^. Since sucrose permeability is low, and relative K^+^/cAMP and K^+^/Lucifer yellow permeability ratios are high (Kanaporis et al., 2008), Cx26 HC permeability to organic buffers such as HEPES is lower than that to Na^+^, and therefore the data on H^+^ equivalents/Na^+^ permeability ratio is an underestimate of the true H^+^/Na^+^ ratio.

Other simple assays that we have employed are “traditional” rapid-filtration assays (Bao et al., 2004c). These are possible because the permeabilities of HCs to “large” hydrophilic solutes, including second messengers (e.g., ATP, cAMP, IP3) is not very high and equilibration between intra- and extraliposomal spaces in HCs containing ~1 HC occurs in >10 s (Kanaporis et al., 2008; Fiori et al., 2012).

## Regulation of Cx43 hemichannels by phosphorylation

Phosphorylation of Cx43 plays a critical role in gap-junction remodeling, and plasma-membrane HC opening in response to ischemic damage in the brain, heart and kidney is likely linked to Cx43 dephosphorylation (Li and Nagy, 2000; Contreras et al., 2002; Hawat et al., 2010; Duffy, 2012). Following phosphorylation of Ser368 by PKC the electrical cell-to-cell coupling (mediated fluxes of small inorganic univalent ions though GJCs) is maintained, whereas the selectivity of chemical coupling (larger hydrophilic solutes) generally decreases, but with an altered selectivity; permeability to negatively-charged solutes decreases, but that to positively-charged ones increases (Kwak et al., 1995; Lampe et al., 2000; Bao et al., 2004a,c; Ek-Vitorin et al., 2006). Single-channel studies have shown that stimulation of PKC decreases the frequency of the dominant of Cx43 GJCs conductance state (~100 pS), favoring a lower conductance state (~50 pS) (Moreno et al., 1992, 1994; Lampe et al., 2000; Ek-Vitorin et al., 2006).

A consequence of Ser368 HC dephosphorylation is an increase in the permeability to large hydrophilic solutes that may cause cell damage due to losses of essential metabolites and second messengers (e.g., glutathione, cAMP, IP3) and/or perhaps influx of Ca^2+^, whereas phosphorylation of GJCs at Ser368 decreases cell-to-cell chemical coupling, which could minimize spreading of the damage to healthy neighboring cells. Uncoupled HCs have been shown to exist in several cell types, and activation of large non-selective Cx43 HCs during ischemia may overwhelm the normal membrane-transport mechanisms and alter intracellular composition, contributing to cell injury. This notion is supported by data on cardiomyocytes, astrocytes, and renal proximal tubule cells that show Cx43 HC activation by ATP depletion (John et al., 1999; Li and Nagy, 2000; Li et al., 2001; Contreras et al., 2002; Vergara et al., 2003b; Hawat et al., 2010). One possibility is that ATP depletion activates the Cx43 HCs by decreasing their phosphorylation state, although other possibilities have been proposed (Kwak et al., 1995; Lampe and Lau, 2000; Lampe et al., 2000; Li and Nagy, 2000; Contreras et al., 2002; Bao et al., 2004a; Retamal et al., 2007b,c; Hawat et al., 2010).

There is significant information available on the role of changes in Cx43 phosphorylation in the heart during cardiac ischemia/hypoxia. The overall effect is a decrease in dephosphorylation that can be a consequence of ATP depletion and/or increased activity of protein phosphatase 1A (Solan and Lampe, 2009; Miura et al., 2010; Duffy, 2012). The changes in Ser368 phosphorylation state are complex. Several constitutively phosphorylated serines are dephosphorylated, including Ser365 (Solan et al., 2007; Solan and Lampe, 2009). Dephosphorylation of Ser365 could be potentially important because its phosphorylation prevents the Ser-368 phosphorylation by PKC (Solan et al., 2007). The combination of Ser365 dephosphorylation and he increase in heart PKC activity in response to ischemia/hypoxia can account for the increase in Ser368 phosphorylation in spite of the overall decrease in Cx43 phosphorylation (Ek-Vitorin et al., 2006; Solan et al., 2007; Solan and Lampe, 2009; Marquez-Rosado et al., 2012). The increase in S368 phosphorylation occurs at the intercalated discs, but Cx43 phosphorylated at S368 has not been found outside the intercalated discs (Ek-Vitorin et al., 2006; Solan et al., 2007; Solan and Lampe, 2009; Marquez-Rosado et al., 2012). Therefore, it seems that in cardiac ischemia total Cx43 expression decreases, but S368-phosphorylated Cx43 increases at the intercalated discs, and Cx43 dephosphorylated at S368 is present in the lateral membranes, probably including HCs. The resulting effects would be a reduction in cell-to-cell coupling at the intercalated discs (minimizing the spread of the damage), and an increase in HC activity that can contribute the ischemic damage.

## Regions of the Cx43 C-terminal domain that are essential for the hemichannel regulation by PKC-mediated phosporylation

The Cx43 CTD can be phosphorylated by several kinases, including PKA, PKC, p34cdc2/cyclin B kinase (p34cdc2), casein kinase 1 (CK1) and pp60src kinase (src), with effects on GJC and HC permeabilities, as well as trafficking, assembly and degradation (Solan and Lampe, 2009). The effects of phosphorylation by these kinases are varied, but here we will concentrate on PKC-mediated phosphorylation of Cx43.

It has been established that the effect of PKC-mediated phosphorylation of Cx43 depends on the presence of the CTD (Solan and Lampe, 2009). A number of low-resolution electron microscopy structures of Cx43 GJCs are available (Unger et al., 1997, 1999a,b; Yeager, 1998). However, these structures do not provide information on the CTD because it had to be removed to improve crystal resolution (Unger et al., 1997, 1999a,b; Yeager, 1998; Cheng et al., 2003). There are NMR structures of the isolated Cx43 CTD (Sorgen et al., 2002, 2004; Hirst-Jensen et al., 2007; Solan et al., 2007; Grosely et al., 2010), but it is presently unclear how closely they resemble the native CTD conformation in the full-length Cx43. One approach to start addressing the specifics of the mechanism is to determine which regions of the CTD are essential for the effect of PKC. To address this question, we performed functional studies in frog oocytes expressing HCs formed by mutants of Cx43 where regions of the CTD were deleted (Bao et al., 2004a). The functional assay consisted of measuring the increase in the uptake of the HC-permeable carboxyfluorescein in response to PKC inhibitors. In Cx43, the CTD extends from approximately E227, and consists of ~155 amino acids. In HCs formed by a Cx43 with deletion of ~60% of the CTD PKC inhibitors still increased carboxyfluorescein uptake. The absence of influence of this large CTD deletion on the response to PKC was directly confirmed in equivalent experiments on purified HCs reconstituted in liposomes (Bao et al., 2004c). The CTD deletion included sequences before and after the Pro-rich region that is essential for the inhibitory effect of lowering intracellular pH and phosphorylation by tyrosine kinases (Warn-Cramer et al., 1996, 1998; Calero et al., 1998; Warn-Cramer and Lau, 2004). Further deletion of the Pro-rich region in our deletion mutant abolished the effect of PKC inhibitors, suggesting a critical role of the Pro-rich region, and therefore a similarity between the effects of acidification and PKC (Bao et al., 2004a). However, replacing of two prolines essential for the pH effect with alanines had no influence on the effect of PKC inhibitors (Bao et al., 2004a). Moreover, replacing 11 amino acids of the Pro-rich region with a 10-Gly linker also had no effect on the response to PKC inhibition (Bao et al., 2004a). Therefore, the mechanisms of changes in HC permeability by Tyr kinases and pH changes, on one side, or by PKC-mediated phosphorylation, on the other, are different. It is still unknown whether the absence of effect of PKC inhibition in the large CTD deletion that included the Pro-rich region is due to the inability of the CTD end (where Ser368 is located) to approach a target region of Cx43. In this context, a ball-and-chain mechanism has been proposed to explain the closure of the Cx43 HC pore in response to intracellular acidification and activation of insulin receptor tyrosine kinase; in this mechanism, the CTD would act as the “ball” that interacts with the C-terminal half of the intracellular loop and occludes the pore (Homma et al., 1998; Delmar et al., 2004; Warn-Cramer and Lau, 2004).

## Identification of the PKC target residue

The observations described above indicate that the CTD is essential for the regulation of HC permeability by PKC-mediated phosphorylation and that the majority of the amino acids in the CTD are not involved in the effect of PKC. It has been established that PKC phosphorylates Cx43 CTD serines, and that the phosphorylated residues are Ser368 and Ser372, neighboring residues near the end of the CTD (Bao et al., 2004a,c; Solan and Lampe, 2009). Replacement of these two residues with alanine individually or in combination indicates that Ser368 is the essential residue for the effect of PKC inhibitors in frog oocytes (Bao et al., 2004a). This is consistent with previous mutagenesis studies in mammalian cells (Lampe et al., 2000) and was directly corroborated by us using purified Cx43 HCs reconstituted in liposomes (Bao et al., 2004c). With this preparation, we first showed that purified Cx43 dephosphorylated by alkaline phosphatase is permeable to organic hydrophilic probes, including sucrose, and that phosphorylation by PKC *in vitro* produces sucrose-impermeable HCs. Then, we showed that this effect of PKC on purified HCs is absent in a Cx43 mutant where S368 was replaced with Ala. The HCs formed by the Cx43-Ser368A mutant are constitutively permeable to sucrose and carboxyfluorescein and do not respond to PKC (Bao et al., 2004a,c). These studies indicate that the effect of PKC on “large” solutes is direct (does not require regulatory intermediate steps), and that the effect is due to phosphorylation of Ser368.

## Conformational changes associated with the regulation by PKC

Since phosphorylation of Ser368 is responsible for the decrease in permeability of Cx43 HCs, phosphorylation of this target residue should elicit a conformational change in the HCs. The nature of this change is still unresolved. As mentioned earlier, a ball-and-chain (or particle-receptor) mechanism has been proposed to explain the closure of the HC pore in response to intracellular acidification and activation of insulin receptor tyrosine kinase (Homma et al., 1998; Delmar et al., 2004; Warn-Cramer and Lau, 2004). Although phosphorylation by PKC does not close the Cx43 HC pore completely (Moreno et al., 1992, 1994; Lampe et al., 2000; Ek-Vitorin et al., 2006), as v-Src and MAPK phosphorylation do (Kim et al., 1999; Cottrell et al., 2003; Warn-Cramer and Lau, 2004), a similar mechanism, but with partial pore closure, is still a possibility. Unfortunately, experimental data addressing the molecular mechanism of regulation of Cx43 GJCs and HCs is largely missing. In a recent study it was shown that mutation of Ser368 and Ser372 to Asp elicits conformational changes in a Cx43 fragment consisting of M4 and the CTD solubilized in detergent (Grosely et al., 2013). The percentage of α-helical structure, of the wild-type fragment, as determined from circular dichroism spectra, increased by lowering the pH from 7.5 (~30%) to 5.8 (~45%), whereas the PKC-phosphorylation-mimicking mutant had an intermediate α-helical content (~40%) that was pH independent. Significant changes in chemical shifts in the PKC mutant fragment were detected by NMR spectroscopy for 14 residues. This is currently the only study where conformational changes were evaluated at the amino-acid level. Assuming that 100% of M4 in the M4-CTD fragment is α helical, it seems that 10–30% of the wild-type CTD is structured (depending on the pH), i.e., the majority is unstructured. In fact, it has been proposed that the CTD is an intrinsically disordered domain, where the phosphate can inhibit binding of the CTD to molecular partners directly, or indirectly, by altering the conformational preference of the disordered region. The latter is a potential mechanism for the change in Cx43 HC permeability.

Although interesting, it is presently unclear whether the structure of the CTD fragments is representative of that in the full-length Cx43. It is possible that interactions of the CTD with other regions of Cx43 result in a structure different from that of the isolated fragments. In this context, although not definitive, our studies suggest a more structured PKC-phosphorylated CTD of purified Cx43 reconstituted in liposomes (Bao et al., 2004c). We performed limited trypsin-proteolysis, and identified digestion products by immunoblotting using an antibody against the Cx43 CTD. We found trypsin-resistant CTD fragments containing the antibody epitope in digests of proteoliposomes containing PKC-phosphorylated Cx43, but not in those of dephosphorylated Cx43 or the Cx43-S368A phosphorylated by PKC. Based on their mobility, the trypsin-resistant fragments correspond to the complete CTD and the CTD plus the last two transmembrane segments. The protection of the entire CTD following phosphorylation of Ser368 would be an unlikely effect in an intrinsically disordered protein, however, it cannot be ruled out since exposure to cleavage sites is not the only factor determining sensitivity to trypsin digestion, but conformational kinetics also plays a role. Independently on the structure of the CTD in full-length Cx43, we also observed a conformational change in response to phosphorylation of Ser368 in purified Cx43 solubilized in detergent. We found that phosphorylation decreases tryptophan fluorescence and produces a blue-shift of the emission peak, suggesting that one or more tryptophans are in a more hydrophobic environment when the protein is dephosphorylated (Bao et al., 2004c). In summary, it has been established that phosphorylation of Ser368 elicits a conformational change of Cx43, but the exact nature of the change and how it relates to the alteration in permeability are unknown.

## Phosphorylation of SER368 produces a partial closure of the Cx43 hemichannel

**Figure 4:**
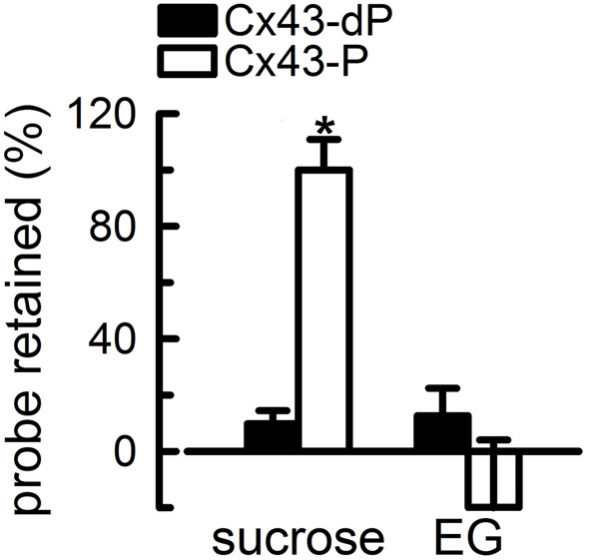
**Partial closure of Cx43 hemichannels by PKC-mediated phosphorylation**. The proteoliposomes were loaded with radiolabeled probes, and the % retention was measured after gel filtration. The proteoliposomes contained HCs formed by either Cx43-dP or Cx43-P. Reconstitution was at an average of 2.3 HCs/liposome. Data are means ± s.e.m. of 4–7 experiments. The asterisk denotes *P* < 0.05 compared with proteoliposomes containing Cx43-dP HCs. Modified from Bao et al., 2007.

In spite of the minimal knowledge on the conformational changes that result from phosphorylation of Ser368, significant progress has been made on mechanistic aspects of the effect of phosphorylation on the purified HCs. One key finding is that contrary to the case of cytoplasmic acidification and MAPK-mediated phosphorylation (Burt and Spray, 1988; Kim et al., 1999; Delmar et al., 2004; Warn-Cramer and Lau, 2004), the closure of the HC pore in response to phosphorylation of Ser368 is partial (Bao et al., 2007). We obtained direct evidence for this in studies on purified HCs reconstituted in unilamellar liposomes (Bao et al., 2007). Phosphorylation of Ser368 by PKC abolishes sucrose permeability of reconstituted Cx43 HCs. However, phosphorylated HCs were still permeable to ethyleneglycol (*Mr* 62 vs. 342 for sucrose) (Figure 4). This observation indicates that phosphorylation by PKC decreases the cross-sectional area of the Cx43 HC pore, but a significant opening remains to allow for ethyleneglycol transport. Since the hydrodynamic radius of ethyleneglycol of 4.4 Å is larger than that of hydrated K^+^ and Cl^−^ (~3.3 Å), it seems likely that PKC-phosphorylated HCs are still permeable to these ions. These results can explain why activation of PKC reduces dye transfer, but has no major effect on cell-to-cell gap-junction currents (Moreno et al., 1992; Takens-Kwak and Jongsma, 1992; Moreno et al., 1994; Kwak et al., 1995; Lampe et al., 2000; Ek-Vitorin et al., 2006).

As mentioned under “regulation of Cx43 HCs by phosphorylation,” stimulation of PKC decreases the frequency of the dominant conductance state of Cx43 GJCs of ~100 pS, favoring a lower conductance state of ~50 pS (Moreno et al., 1992, 1994; Lampe et al., 2000; Ek-Vitorin et al., 2006). It is possible that these lower conductance channels are formed by the phosphorylated HCs permeable to ethyleneglycol, but not sucrose. However, the level of Cx43 phosphorylation in cells' studies is undefined, and there is no simple correlation between single-HC conductance and permeability to large hydrophilic solutes by different connexin isoforms (Harris, 2001; Nielsen et al., 2012). Independently of these uncertainties, PKC-mediated phosphorylation of Cx43 could reduce fluxes of organic hydrophilic solutes such as ATP, cAMP, and IP3 (*Mr* 300–700) without major effects on small-inorganic ion fluxes and cell-to-cell electrical coupling.

## Phosphorylation of all subunits is necessary to abolish Cx43 hemichannel permeability to “large” hydrophilic solutes

The observations that HCs formed by dephosporylated Cx43 (Cx43-dP) are permeable to sucrose, but those formed by Cx43 phosphorylated by PKC (Cx43-P) are not (Bao et al., 2004c), provided us with a tool to determine the number of subunits that need to be phosphorylated at Ser368 to abolish HC sucrose permeability. It is relatively simple to study the function of GJCs and HCs that are fully dephosphorylated at Ser368 by expressing the Cx43-S368A mutant in cell lines devoid of endogenous connexins. However, it is hard to express HCs with a known number of subunits phosphorylated at Ser368. Fortunately, Cx43 HC of controlled subunit composition can be generated *in vitro* from purified Cx43. As mentioned under “Charaterization of purified connexin hemichannels,” purified Cx43 (but not Cx26) solubilized in detergent forms HCs where the connexins (subunits) exchange (Bao et al., 2007). This allowed us to generate and reconstitute Cx43 HCs of controlled average subunit composition.

We demonstrated exchange of subunits and determined subunit composition in semi-quantitative and quantitative manners, using size-exclusion chromatography and luminescence resonance energy transfer (LRET), respectively (Bao et al., 2007). For the former experiments, we mixed Cx43 and Cx43-EGFP (Cx43 with an enhanced green fluorescent protein fused to the C-terminus of the CTD). Each Cx43 fused to EGFP adds ~26 kDa, increasing the hydrodynamic size of the HCs significantly, and in proportion to the average number of Cx43-EGFP subunits in the HCs. Although this method is sensitive, it lacks accuracy to discriminate between, for example, HCs containing 3 or 5 Cx43-EGFP subunits. This was solved using LRET. With this spectroscopic technique, we measured energy transfer between donor and acceptor LRET probes reacted to Cx43 cysteines.

**Figure 5:**
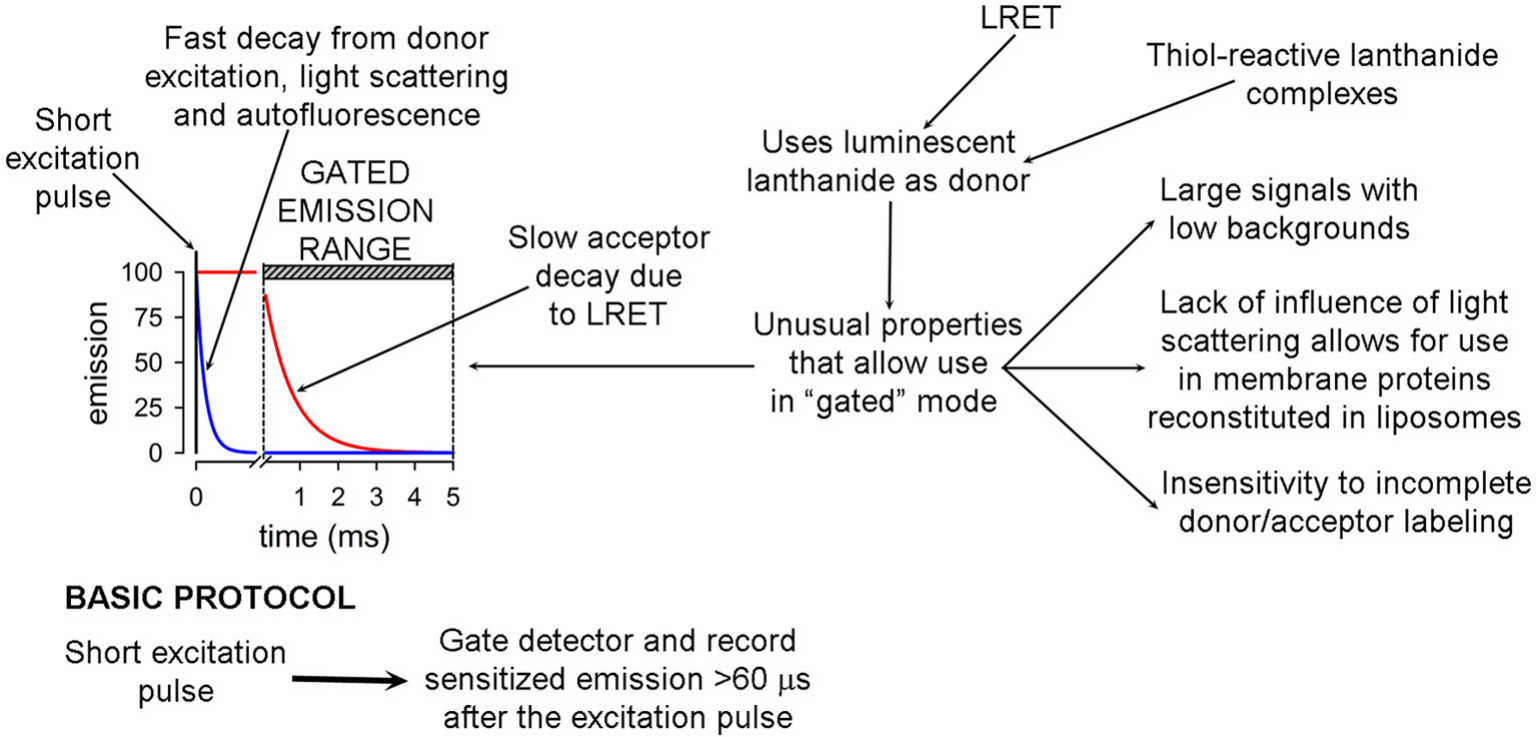
**Basic principles and advantages of luminescence resonance energy transfer**. The basic gated-mode LRET protocol that takes advantages of the long lifetime of the donor compared to the fluorophore acceptor is summarized at the bottom.

LRET measures energy transfer from the lanthanides Tb^3+^ or Eu^3+^ to fluorescent acceptor molecules (Selvin, 2002). It has atomic-resolution and high sensitivity, and can also provide dynamic information on conformational changes in the millisecond to minute time frame (Posson et al., 2005; Posson and Selvin, 2008; Rambhadran et al., 2011; Zoghbi et al., 2012; Cooper and Altenberg, 2013; Zoghbi and Altenberg, 2013). Therefore, it will be useful for future studies of the conformational changes elicited by phosphorylation of Ser368. LRET has many advantages over traditional FRET (Selvin, 2002). Its basic principles and advantages have been described in detail, and are presented in Figure 5. The main advantages derive from the long lifetime of the Tb^3+^ and Eu^3+^ excited states, which allows for acquisition of long-lifetime donor emission in a gated mode (i.e., delaying acquisition, generally for 60–200 μs). Gated acquisition minimizes the light scattering effects of structures such as detergent micelles and liposomes, and results in minimal background with high signal-to-noise ratio. In addition, LRET-based distances can be measured in the 25–120 Å range and are more accurate than those based on FRET because donor and sensitized acceptor emission are unpolarized, and therefore uncertainty about κ (geometric factor related to the relative orientation of the donor and acceptor transition dipoles) is not an issue (Selvin, 2002; Zoghbi et al., 2012). Another major advantage is that the sensitized emission lifetime is independent of the labeling stoichiometry because long-lifetime acceptor emission can arise only from LRET (Selvin, 2002; Bao et al., 2007), i.e., labeling stoichiometry affects the intensity of the signal, but not its lifetime. Finally, the “atomic-like” lanthanide emission (sharp peaks with dark regions between them) allows for measurements of acceptor emission without contamination from the donor emission (Selvin, 2002; Zoghbi et al., 2012; Cooper and Altenberg, 2013).

**Figure 6:**
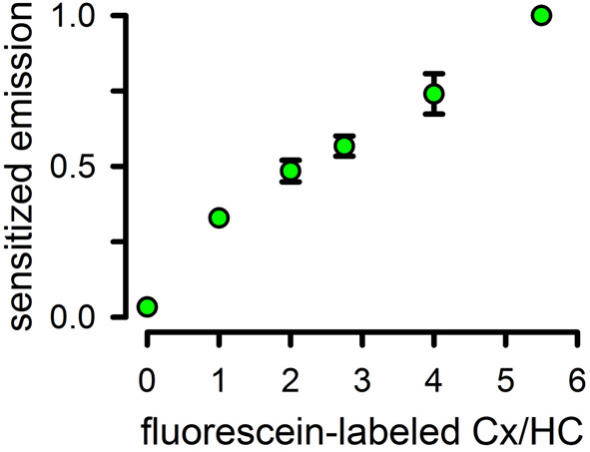
**Use of luminescence resonance energy transfer to assay hemichannels subunit composition**. Sensitized fluorescence emission as a function of the average number of fluorescein-labeled subunits/HC. Data were normalized to the peak value of 1 Tb/5.5 fluorescein-labeled preparation and are means ± s.e.m. of 7–9 experiments. Reconstitution was at an average of 0.8 HCs/liposome. All values are statistically different from the previous one (*P* < 0.001), except for that at the 3/3 ratio. Modified from Bao et al., 2007.

To test LRET on Cx43 HCs, we labeled Cx43 with either fluorescein maleimide as acceptor or Tb^3+^-DTPAcs124-EMCH as donor, by incubation with a 10-fold molar excess of the thiol reagents for 2 h at 4°C. Cx43 has 9 cysteines (Bao et al., 2007). Six are located in the extracellular loop, and are likely to form intramolecular disulfide bonds (Foote et al., 1998; Maeda et al., 2009). These may not be accessible for labeling. The remaining 3 cysteines are located in the CTD, and may be available for labeling. The labeling stoichiometry was 3 probe molecules/HC, suggesting that all CTD cysteines are solvent accessible. After labeling, we mixed fluorescein-labeled, Tb^3+^-labeled and unlabeled Cx43 in different proportions, but always using a low proportion of Tb^3+^-labeled Cx43 (0.5 mol/HC). Under these conditions, most HCs have either one Tb^3+^-labeled subunit or none, with only ~8% containing more than one donor-labeled subunit (calculated from the binomial distribution). Under these conditions, considering that essentially all subunits assemble as functional HCs, the fluorescein emission intensity due to energy transfer from Tb^3+^ (long lifetime sensitized fluorescein emission) depends on the number of fluorescein-labeled subunits/HC, and can be used to determine the HC subunit composition (Figure 6). Details and validation of the methodology have been published (Bao et al., 2007). The LRET-based method is very accurate; it can discriminate between HCs containing ± 1 acceptor-labeled subunits (Bao et al., 2007). In addition, measurements can be obtained from HCs in liposomes, which allows for parallel determinations of HC subunit composition and function.

**Figure 7:**
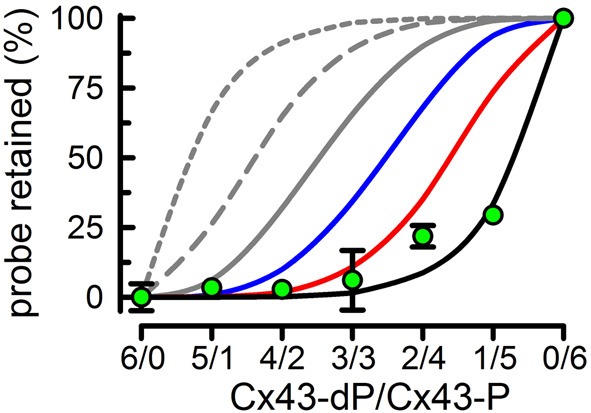
**All subunits have to be phosphorylated by PKC to abolish Cx43-HC sucrose permeability**. Effects of varying Cx34-dP/Cx43-P average ratios on the % sucrose-permeable proteoliposomes preloaded with radiolabeled sucrose. Data are means ± s.e.m. of 4–7 experiments. Lines represent the expected % (calculated from the binomial distribution) if the number of Cx43-P subunits necessary to render the HC impermeable to sucrose are ≥1 (gray, short dash), ≥2 (gray, long dash), ≥3 (gray, solid), ≥4 (blue), ≥5 (red), or 6 (black). Modified from Bao et al., 2007.

After demonstrating that the average subunit composition of reconstituted HCs is that of the subunit mixes in detergent, we went back to the original question of the number of subunits that have to be phosphorylated by PKC to abolish HC sucrose permeability. To answer the question, we performed experiments with 0.8 HC/liposomes on average. In this way, very few liposomes contain more than one HC, and therefore the % of sucrose-permeable liposomes depends on whether the HC in that liposome is permeable or not. Also, if subunits form HCs randomly, the subunit composition of the HCs will follow the binomial distribution, e.g., for a 3/3 mixture of Cx43-dP/Cx43-P the most frequent HCs will contain 3 Cx43-dP and 3 Cx43-P subunits (~31%), with lower frequencies of 2/4 and 4/2 (~23% each), 1/5 and 5/1 (~9% each), and 0/6 and 6/0 (~2% each). Proteoliposomes with HCs formed only by Cx43-P are impermeable to sucrose, whereas those formed by Cx43-dP alone or Cx43-dP/Cx43-P mixtures of 5/1, 4/2 or 3/3 were permeable to sucrose (Bao et al., 2007). Therefore, 3 Cx43-P subunits/HC are insufficient to abolish sucrose permeability. With more than 3 Cx43-P subunits/HC, the percentage of sucrose-impermeable liposomes becomes significant and proportional to the number of Cx43-P subunits (Bao et al., 2007). However, even for an average of 5 Cx43-P/HC, the % of sucrose-impermeable liposomes was only ~30%, even though ~83% of the Cx43 is Cx43-P. If 5 Cx43-P/HC were sufficient to produce sucrose-impermeable HCs, the expected % of sucrose-impermeable liposomes in the 1/5 Cx43-dP/Cx43-P HCs would be ~74%. This is the sum of the proteoliposomes containing 5 (~40%) and 6 (~34%) Cx43-P subunits according to the binomial distribution. Since this value is more than twice the value measured (~30%), but very close to the percentage of proteoliposomes containing 6 Cx43-P subunits (~34%), it appears that all 6 HC subunits must be phosphorylated to abolish sucrose permeability (Figure 7) (Bao et al., 2007). It is important that the transport-specific sedimentation assay used to determine whether the liposomes were permeable or impermeable to sucrose, provides steady-state information on the permeability cut-off, but not information on the rates of permeation. Therefore, our results cannot rule out that partial phosphorylation of Cx43 HCs decreases sucrose permeability, but show that complete phosphorylation is needed to abolish sucrose permeability. Studies of the kinetics of transport such as those that we have developed recently (see “Functional analysis of purified and reconstituted connexin hemichannels”) can be used to address this issue.

## Summary and perspectives

Purified HCs constitute an excellent preparation for functional and structural studies of Cx26 and Cx43 HCs. Although still under development, there are assays to analyze the function and structure of these HCs under “basal” conditions, as well as to evaluate functional and structural changes in response to regulatory factors. Focusing on the regulation of Cx43 by phosphorylation, a number of significant findings have been made using purified HCs. These include: (a) the effect of PKC is direct on the HCs (no intermediate regulatory proteins), (b) phosphorylation of S368 is the only event needed for the alterations in permeability produced by PKC-mediated phosphorylation, (c) phosphorylation of S368 results in conformational changes of Cx43 HCs demonstrated by a blue shift of the peak emission of the native tryptophans and a protection of the CTD from trypsin digestion, (d) phosphorylation of S368 produces a partial closure of the HC pore that alters its selectivity: it abolishes sucrose permeability, but not that to the smaller hydrophilic solute ethyleneglycol, (e) to abolish sucrose permeability all six HC subunits have to be phosphorylated; 5 of the 6 is not sufficient. All indications are that important advances will be made in the near future on the molecular mechanism of HC regulation by correlating functional effects and structural changes determined by combining X-ray crystallography and spectroscopic techniques such as LRET.

### Conflict of interest statement

The authors declare that the research was conducted in the absence of any commercial or financial relationships that could be construed as a potential conflict of interest.
